# SARS-CoV-2 Infection Can Lead to an Increase in Tacrolimus Levels in Renal Transplant Patients: A Cohort Study

**DOI:** 10.3389/ti.2022.10127

**Published:** 2022-03-21

**Authors:** Christopher G. Chalklin, Georgios Koimtzis, Usman Khalid, Eliot Carrington-Windo, Doruk Elker, Argiris Asderakis

**Affiliations:** ^1^ Cardiff Transplant Unit, University Hospital of Wales, Cardiff and Vale University Health Board, Cardiff, United Kingdom; ^2^ College of Medicine, Infection and Immunity, Cardiff University, Cardiff, United Kingdom

**Keywords:** SARS-CoV-2, kidney transplantation, immunosuppression, tacrolimus, AKI, tacrolimus levels

## Abstract

The aim of this study is to evaluate the effect of SARS-CoV-2 infection on serum tacrolimus levels. Tacrolimus levels of 34 transplant patients diagnosed with SARS-CoV-2 in 2020 were compared with their pre-infection values and those of a control group with alternative infections. 20 out of 34 (59%) had high levels. At diagnosis, median tacrolimus level in the SARS-CoV-2 cohort was 9.6 μg/L (2.7–23) compared to 7.9 μg/L in the control group (*p* = 0.07, 95% CI for difference −0.3–5.8). The ratio of post-infection to pre-infection tacrolimus values was higher in the SARS-CoV-2 group (1.7) compared to the control group (1.25, *p* = 0.018, 95% CI for difference 0.08–0.89). The acute kidney injury rate was 65% (13 of 20) in SARS-CoV-2 patients with a level >8 μg/dl, compared to 29% (4 of 14) in those with lower levels (*p* = 0.037). Median length of stay was 10 days among SARS-CoV-2 infected patients with high tacrolimus levels compared to 0 days in the rest (*p* = 0.04). Four patients with high levels died compared to 2 in the control group. Clinicians should be aware of this potential effect on tacrolimus levels and take appropriate measures.

## Graphical Abstract

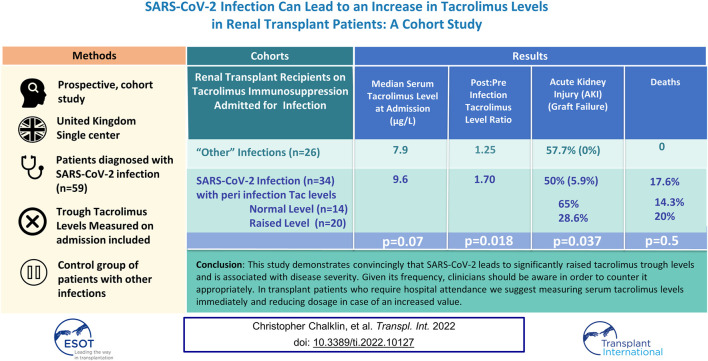

## Introduction

Severe acute respiratory distress syndrome coronavirus 2 (SARS-CoV-2) has emerged as worldwide pandemic (1). Risk factors for mortality include advanced age, chronic kidney disease, diabetes mellitus, obesity, cardiovascular disease, history of malignancy and chronic immunosuppression (1–5). As a result, transplant patients are more vulnerable with a higher mortality rate which differed amongst reports, prior to vaccination, between 11% and 50% (6–8).

British and American guidelines recommend immunosuppression modification during the treatment of SARS-CoV-2 infection in transplant patients (9, 10), mainly consisting of withdrawal of one or more immunosuppression drugs. Most commonly, antimetabolites such as mycophenolate derivatives are discontinued while other immunosuppressants such as calcineurin inhibitors are administered at a lower dose or occasionally stopped (6, 7). Some though, suggest that calcineurin inhibitors may also inhibit the replication of coronaviruses such as SARS-CoV-2 (11–13) although this is not the prevalent view currently.

Nevertheless, until now, there has been no data published on the effect that SARS-CoV-2 infection has on serum levels of immunosuppression medication.

During the “first wave” of the pandemic we observed a number of renal transplant patients with unusually high serum tacrolimus levels, therefore we set out to investigate if the presence of SARS-CoV-2 infection was associated with increased calcineurin inhibitor levels in the cohort of transplant patients affected by SARS-CoV-2 in Wales, and whether this had contributed to acute kidney injury (AKI) and patient outcome. We were also interested to see if this increase was more pronounced from the one seen in other acute inflammatory conditions or infections (14, 15).

## Materials and Methods

This is a cohort study performed by maintaining a prospective database of transplant patients cared for by the Cardiff Transplant Unit who were diagnosed with SARS-CoV-2 infection between 1st March 2020–31st December 2020 since, during this period, outcomes were not affected by vaccination. Transplant patients who presented to the emergency department in any of the South and Mid Wales hospitals or to the transplant telephone service with a presumed diagnosis of SARS-CoV-2 and had a positive result on real-time polymerase chain reaction (PCR) assay were included. One single transplant clinician collated the data prospectively and dedicated members of the team communicated with the treating team if patients were admitted to any of the surrounding hospitals or called them at home if not. Patients developing “classical” SARS-CoV-2 symptoms were initially directed to self-isolate, however, if unwell or upon deterioration to present to their local hospital. Data and outcomes of those more severely affected transplant recipients along with waiting list patients during the first wave of the disease from our region has been published (16).

Cases were recorded alongside demographics, symptoms at diagnosis, serum tacrolimus level at diagnosis and the previous visits, hospital admission, intensive care admission and 30-day outcomes including mortality. No patients were lost to follow-up.

Patients were on different immunosuppression regimes. The target range for tacrolimus levels in this unit is generally between 5 and 8 μg/L. Once patients were diagnosed with SARS-CoV-2 infection, their immunosuppression medication was reviewed. Patients who were on tacrolimus were identified, and those who had their trough serum level measured at the time or close (±2 days) to the diagnosis were included in the final analysis. Patients who did not have their level measured at the appropriate time or who were not taking tacrolimus were excluded from this analysis. The presence (or not) of diarrhoea was also recorded as a potential symptom of SARS-CoV-2 infection due to its effect on tacrolimus absorption from the gut.

Once the cohort of patients was identified, their serum tacrolimus levels were examined and the mean of the three most recent levels for each patient immediately prior to infection was calculated and represented their pre-infection level. Following admission, mycophenolate derivatives were withheld as a standard practice.

A “control” group of patients was identified by collecting the data of all sequential admissions with sepsis to our unit over a period of 1 year. Diarrheal illnesses were excluded as this is well known to affect serum tacrolimus concentrations (17).

The same data was collected for these patients for comparison with patients presenting with SARS-CoV-2 infection.

Median values were compared with Mann Whitney test. In addition the ratio of tacrolimus level post- and pre-SARS-CoV-2 infection was generated for each patient and was compared with the respective ratio of patients who had other types of infection by performing a t-test without presumption of equal variances. Analysis was performed using IBM-SPSS version 25.0 software.

This article was prepared following the STROBE statement-checklist.

## Results

During this period, 59 transplant patients were diagnosed with SARS-CoV-2 infection. 52 of were taking tacrolimus and 34 had their trough serum tacrolimus level measured at the time of diagnosis (including all those admitted). Of these 34 patients, 20 (38.4% of infected patients on tacrolimus and 58.8% of those with available trough levels ±2 days to diagnosis) had a value above 8 μg/L and 14 had a value within the unit’s ‘normal range’. The 18 patients who had not had their tacrolimus level measured were patients with mild symptoms that did not attend any healthcare facility in person and were advised to self-isolate.

Median age at diagnosis for these patients was 54.5 years (range 25–80), 23 patients (67%) were male and 33 (97%) were from a white European background*.* Median time between diagnosis and transplantation was 82.5 months (range 1–317)*.* Five patients (14.7%) experienced diarrhoea (with another three developing it later). 22 patients (64.7%) required hospital admission with 4 of them (11.8%) requiring escalation to an intensive care setting*.* 17 patients (50%) suffered graft dysfunction (AKI) and 2 patients (5.9%) had graft failure requiring return to dialysis. Four patients (11.8%) died during hospital admission due to COVID-19 infection*.* All deaths occurred within the group of patients with graft dysfunction, but the patients who suffered graft loss survived. A summary of the characteristics of the patients in this cohort is provided in Table 1.

**TABLE 1:** Characteristics of transplant recipients with available serum Tacrolimus levels at the time of diagnosis with SARS-CoV-2 compared to a control group of other infected patients who required hospital admission.

Group		SARS-CoV-2 (*n* = 34)	Control (*n* = 26)
Age (years)	Median (range)	54.5 (25–80)	55 (25–83)
Sex	Male	23 (67%)	14 (53.8%)
	Female	11	12
Ethnicity	White European	33 (97%)	24 (92.3%)
	East Asian	1	1
	South Asian	0	1
BMI (kg/m^2^)	Median (range)	28.7 (22–41.5)	25.3 (18–39)
Type of transplant organ/donor	Kidney (living donor)	11 (32%)	10 (38.5%)
	Kidney (DBD)	14 (41%)	10 (38.5%)
	Kidney (DCD)	8 (23.5%)	5 (19%)
	Simultaneous pancreas and kidney	1 (3%)	1 (3.8%)
Transplant to infection diagnosis (months)	Median (range)	82.5 (1–317)	112 (22–328)
Hospital admission status and outcome	Outpatient	12 (35.3%)	—
	Inpatient	22 (64.7%)	26
	Intensive care unit	4 (11.8%)	0
	Graft dysfunction	17 (50%)	15 (57.7%)
	Graft failure	2 (5.9%)	0
	Death	4 (11.8%)	0

The control group consisted of 26 consecutive patients admitted with other infections to the transplant unit over 1 year period. Urinary tract infections were most common and affected 21 (80.8%) of the admitted patients. Other infections documented were non-SARS-CoV-2 respiratory infections (three cases, 11.5%), biliary (one case, 3.8%) and cryptococcal meningitis (one case, 3.8%). Median age at diagnosis was 55 years (range 25–83). Median time from transplantation to infection was 112 months (range 22–328). All patients were cared for on the transplant ward (level 1 and 2 care). No deaths or graft failure occurred in this group, 15 (57.7%) presented with an acute kidney injury.

The range of trough serum tacrolimus levels at the time of diagnosis for the patients in the SARS-CoV-2 cohort was 2.7–23 μg/L, mean 11.2, median 9.6. As mentioned, 20 of those patients had a level above 8 μg/L. This contrasted with a mean value of 8.5 μg/L and a median of 7.9 μg/L in the control group (Mann-Whitney *p* = 0.07, 95% CI for the difference between medians −0.3–5.8) (Table 2).

**TABLE 2:** The serum tacrolimus levels where higher among SARS-CoV-2 infected patients compared to controls admitted due to other infections.

		SARS-CoV-2 (*n* = 34)	Control (*n* = 26)	
Serum tacrolimus level (µg/L)	Range	2.7–23	2–27.8	*p* = 0.07
	Median	9.6	7.9	95% CI (−0.3–5.8)

**FIGURE 1:**
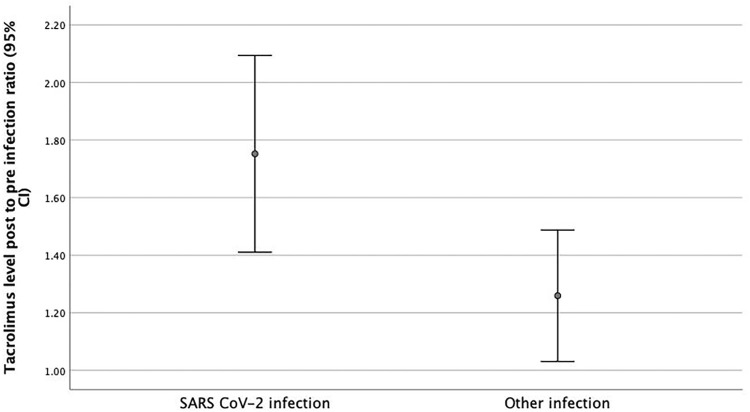
Post to Pre-Infection Tacrolimus Level Ratio. Post to Pre infection Tacrolimus level ratios were higher in patients with SARS- CoV-2 infection (1.7 μg/L) at the time of diagnosis compared to transplant patients admitted with other infections (1.25 μg/L) (*p* = 0.018, mean ratio difference 0.49, 95% CI 0.08–0.89).

To ensure that the observed difference was real, a ratio was calculated of the post-infection tacrolimus trough level to the mean pre-infection value. This ratio was higher in the SARS-CoV-2 cohort (ratio 1.7) compared to the control group (ratio 1.25, *p* = 0.018, mean ratio difference 0.49, 95% CI 0.08–0.89).

(Figure 1) A higher incidence of graft dysfunction (AKI) was found in SARS-CoV-2 infected patients who had tacrolimus levels above 8 μg/dl following infection, 13 out of 20 compared to 4 out of 14 among those with lower levels (*p* = 0.037).

The median length of stay was higher among SARS-CoV-2 infected patients with higher tacrolimus levels (10 days, range 1–44) compared to those who did not have high levels (0 day, range 0–70, *p* = 0.04) although the incidence of ICU admissions was the same. Four of the 20 patients (25%) with high tacrolimus levels died, compared to 2 of the 14 patients with normal tacrolimus levels (*p* = 0.5).

## Discussion

This small study demonstrates convincingly that SARS-CoV-2 leads to significantly raised tacrolimus trough levels and is associated with disease severity.

Acute kidney injury has been recognized as a prominent complication of SARS-CoV-2 infection resulting from an immunological cascade leading to vascular, tubular, and glomerular injury (18). AKI complicates 4.3% of the cases of SARS-CoV-2 that require hospitalization and almost 20% of the critically ill patients require renal replacement therapy (RRT) (18–20). AKI has also been associated with higher mortality rate in critically ill patients with SARS-CoV-2 infection (21). The AKI is multifactorial but a specific proximal tubular injury has been described by Werion et al (22)*.* A novel injury mechanism after SARS-CoV-2 entry, which is based on expression and functional network analysis between *ACE2* and solute channel genes has been considered.

Diarrhoea is a common symptom of SARS-CoV-2 in children (23) and it might affect up to 13.5% of adults (24). SARS-CoV-2 invades the gastrointestinal tract through binding with ACE2 receptors (for which it has 10–20 times higher affinity compared to SARS-CoV-1) (25) causing intestinal permeability changes. Mouse models have shown that ACE2 alterations might be associated with the uptake and imbalance of amino acids and colitis (26).

Five of the 20 patients with high tacrolimus levels had diarrhea, that is well recognized to lead to increased tacrolimus levels (17). The mechanism of increased levels in the rest is still unclear, but it is of unusually high frequency. Whether tacrolimus levels were raised in patients for whom a contemporaneous level was not available is difficult to say. It may be that high levels are associated with more severe disease that prompts hospital attendance or admission. We postulate that increased tacrolimus levels are either the result of decreased transit time in the gastrointestinal tract (small bowel) with increased enterohepatic circulation, being due to further reduction in Pgp levels compared to other infections or a direct effect of the increased permeability following binding of ACE2 receptors.

The current study brings to light another aspect of SARS-CoV-2 infection in transplant patients: tacrolimus-induced nephrotoxicity. In this cohort the serum tacrolimus level was significantly raised compared to previous values in the same patients as indicated by an ‘infection to pre-infection’ ratio of 1.7. Patients with those higher levels were at a higher risk for developing AKI and stayed longer in the hospital. In addition, the post-infection levels of tacrolimus among SARS-CoV-2 infected patients were increased compared to a control (non-SARS-CoV-2) infection group that required admission. It is well known that, in the setting of inflammation, ischemia and shock, P-glycoprotein (PgP) expression in the gut wall may be reduced leading to decreased PgP levels and increased blood tacrolimus trough concentrations up to two times, as it occurred in the present study (14, 15, 27–30). The inclusion of a control group of serious infection that required admission confirms these particularly raised levels truly related to SARS-CoV-2 itself rather than a what is occurring with any severe infection.

SARS-CoV-2 infection could therefore impair renal function in transplant patients both by damaging the kidney directly and by causing drug-induced nephrotoxicity due to higher tacrolimus levels conveying a higher risk of overall morbidity.

As a conclusion, increased serum tacrolimus level is another effect of SARS-CoV-2 infection that has yet to be fully understood. Given its frequency, clinicians should be aware and be vigilant in order to counter it appropriately even in patients not experiencing diarrhoea. In transplant patients who require hospital attendance we suggest measuring serum tacrolimus levels immediately and reducing dosage appropriately in case of an increased value. This study is limited by the small sample size and the unmatched, heterogenous nature of the control group that could have also contributed to the difference in tacrolimus levels. Further research can determine the pathophysiological mechanism involved in this process, alongside careful control group design to gain more insight to it.

## Data Availability

The original contributions presented in the study are included in the article/Supplementary Material, further inquiries can be directed to the corresponding author.

## Abbreviations

AKI, acute kidney injury; ARDS, acute respiratory distress syndrome; AUC, area under the curve; BMI, body mass index; COVID-19, Coronavirus disease 2019; MMF, mycophenolate mofetil; PCR, polymerase chain reaction; PgP, P-glycoprotein; RRT, renal replacement therapy; SARS-CoV-2, Severe acute respiratory distress syndrome coronavirus 2; SPK, simultaneous pancreas and kidney transplant.

## References

[1] Asselah T Durantel D Pasmant E Lau G Schinazi RF. COVID-19: Discovery, Diagnostics and Drug Development. J Hepatol (2021) 74 (1):168–84. 10.1016/j.jhep.2020.09.031 33038433 PMC7543767

[2] Cai R Zhang J Zhu Y Liu L Liu Y He Q. Mortality in Chronic Kidney Disease Patients with COVID-19: a Systematic Review and Meta-Analysis. Int Urol Nephrol (2021) 53:1623–9. 10.1007/s11255-020-02740-3 33389508 PMC7778685

[3] Lunski MJ Burton J Tawagi K Maslov D Simenson V Barr D Multivariate Mortality Analyses in COVID‐19: Comparing Patients with Cancer and Patients without Cancer in Louisiana. Cancer (2021) 127(2):266–74. 10.1002/cncr.33243 33112411

[4] Varikasuvu SR Dutt N Thangappazham B Varshney S. Diabetes and COVID-19: A Pooled Analysis Related to Disease Severity and Mortality. Prim Care Diabetes (2021) 15(1):24–7. 10.1016/j.pcd.2020.08.015 32891525 PMC7456278

[5] Azzi Y Bartash R Scalea J Loarte-Campos P Akalin E. COVID-19 and Solid Organ Transplantation: A Review Article. Transplantation (2021) 105(1):37–55. 10.1097/tp.0000000000003523 33148977

[6] Mahalingasivam V Craik A Tomlinson LA Ge L Hou L Wang Q A Systematic Review of COVID-19 and Kidney Transplantation. Kidney Int Rep (2021) 6(1):24–45. 10.1016/j.ekir.2020.10.023 33163708 PMC7607258

[7] Avery RK. COVID-19 Therapeutics for Solid Organ Transplant Recipients; 6 Months into the Pandemic: Where Are We Now? Transplantation (2021) 105(1):56–60. 10.1097/tp.0000000000003519 33141805

[8] NHSBT ref: SARS-CoV-2 cases in wait list and organ transplant patients (2021). Available from: https://nhsbtdbe.blob.core.windows.net/umbraco-assets-corp/23227/weekly-report-on-covid-19-nhsbt-14-may-2021.pdf (Accessed January 06, 2022).

[9] BTS Guidance on the management of transplant recipients diagnosed with or suspected of having COVID19 (2021). Available from: https://bts.org.uk/wp-content/uploads/2021/01/Clinical-management-of-transplants-and-immunosuppression-22nd-January-2021-FINAL.pdf (Accessed January 06, 2022).

[10] COVID-19 Treatment Guidelines Panel. Coronavirus Disease 2019 (COVID-19) Treatment Guidelines. National Institutes of Health (2022). Available from: https://www.covid19treatmentguidelines.nih.gov/ (Accessed January 06, 2022). 34003615

[11] Andersen KM Mehta HB Palamuttam N Ford D Garibaldi BT Auwaerter PG Association between Chronic Use of Immunosuppresive Drugs and Clinical Outcomes from Coronavirus Disease 2019 (COVID-19) Hospitalization: A Retrospective Cohort Study in a Large US Health System. Clin Infect Dis (2021) 73:e4124–e4130. 10.1093/cid/ciaa1488 33410884 PMC7953980

[12] Lai Q Spoletini G Bianco G Graceffa D Agnes S Rossi M SARS‐CoV2 and Immunosuppression: A Double‐edged Sword. Transpl Infect Dis (2020) 22:22. 10.1111/tid.13404 PMC7361075 32639598

[13] Hochleitner B-W Bösmüller C Nehoda H Steurer W Konigsrainer A Margreiter R Increased Tacrolimus Levels during Diarrhea. Transpl Int (2001) 14:230–3. 10.1007/s001470100331)10.1111/j.1432-2277.2001.tb00050.x 11512055

[14] Bd M. Differential Effect of Diarrhea on FK506 versus Cyclosporine A Trough Levels and Resultant Prevention of Allograft Rejection in Renal Transplant Recipients. Am J Transpl (2002) 2(10):989–92. 10.1034/j.1600-6143.2002.21018.x 12484345

[15] Niemi M. High Plasma Pravastatin Concentrations Are Associated with Single Nucleotide Polymorphisms and Haplotypes of Organic Anion Transporting Polypeptide-C (OATP-C, SLCO1B1). Pharmacogenetics (2004) 14(7):429–40. 10.1097/01.fpc.0000114750.08559.32 15226675

[16] Khalid U Ilham MA Nagaraja P Elker D Asderakis A. SARS-CoV-2 in Kidney Transplant and Waitlisted Patients during the First Peak: The Welsh Experience. Transplant Proc (2021) 53:1154–9. 10.1016/j.transproceed.2020.12.002 33478747 PMC7834026

[17] Bentata Y. Tacrolimus: 20 Years of Use in Adult Kidney Transplantation. What We Should Know about its Nephrotoxicity. Artif Organs (2020) 44(2):140–52. 10.1111/aor.13551 31386765

[18] Chan L Chaudhary K Saha A Chauhan K Vaid A Zhao S AKI in Hospitalized Patients with COVID-19. Jasn (2021) 32(1):151–60. 10.1681/asn.2020050615 32883700 PMC7894657

[19] Liu Y-F Zhang Z Pan X-L Xing G-L Zhang Y Liu Z-S The Chronic Kidney Disease and Acute Kidney Injury Involvement in COVID-19 Pandemic: A Systematic Review and Meta-Analysis. May (2020) 2020. 10.1101/2020.04.28.20083113 PMC7785235 33400721

[20] Alessandri F Pistolesi V Manganelli C Ruberto F Ceccarelli G Morabito S Acute Kidney Injury and COVID-19: A Picture from an Intensive Care Unit. Blood Purif (2021) 50:767–71. 10.1159/000513153 33412548

[21] Werion A Belkhir L Perrot M Schmit G Aydin S Chen Z SARS-CoV-2 Causes a Specific Dysfunction of the Kidney Proximal Tubule. Kidney Int (2020) 98(5):1296–307. 10.1016/j.kint.2020.07.019 32791255 PMC7416689

[22] Mayor S. Covid-19: UK Studies Find Gastrointestinal Symptoms Are Common in Children. BMJ (2020) 370:m3484. 10.1136/bmj.m3484 32895221

[23] Zhao Y Cao Y Wang S Cai K Xu K. COVID-19 and Gastrointestinal Symptoms. Br J Surg (2020) 107(10):e382–e383. 10.1002/bjs.11821 32757447 PMC7436706

[24] Wrapp D Wang N Corbett KS Goldsmith JA Hsieh C-L Abiona O Cryo-EM Structure of the 2019-nCoV Spike in the Prefusion Conformation. Science (2020) 367(6483):1260–3. 10.1126/science.aax090210.1126/science.abb2507 32075877 PMC7164637

[25] Xiao F Tang M Zheng X Liu Y Li X Shan H. Evidence for Gastrointestinal Infection of SARS-CoV-2. Gastroenterology (2020) 158(6):1831–3. 10.1053/j.gastro.2020.02.055 32142773 PMC7130181

[26] Hashimoto T Perlot T Rehman A Trichereau J Ishiguro H Paolino M ACE2 Links Amino Acid Malnutrition to Microbial Ecology and Intestinal Inflammation. Nature (2012) 487(7408):477–81. 10.1038/nature11228 22837003 PMC7095315

[27] Royer B Larosa F Legrand F Gerritsen-van Schieveen P Bérard M Kantelip J-P Pharmacokinetics of Mycophenolic Acid Administered 3 Times Daily after Hematopoietic Stem Cell Transplantation with Reduced-Intensity Regimen. Biol Blood Marrow Transplant (2009) 15(9):1134–9. 10.1016/j.bbmt.2009.04.011 19660728

[28] Eyal S. Histone Deacetylases Inhibition and Tumor Cells Cytotoxicity by CNS-Active VPA Constitutional Isomers and Derivatives. Biochem Pharmacol (2005) 69(10):1501–8. 10.1016/j.bcp.2005.02.012 15857614

[29] Lemahieu W. Cytochrome P450 3A4 and P-Glycoprotein Activity and Assimilation of Tacrolimus in Transplant Patients with Persistent Diarrhea. Am J Transpl (2005) 5(6):1383–91. 10.1111/j.1600-6143.2005.00844.x 15888045

[30] Percy C Hassoun Z Mourad M De Meyer M Beguin C Jadoul M Impact of Acute Infection Requiring Hospitalization on Tacrolimus Blood Levels in Kidney Transplant Recipients. Transplant Proc (2017) 49(9):2065–9. 10.1016/j.transproceed.2017.09.019 29149962

